# Effects and mechanisms of *Salvia miltiorrhiza* Bunge extract on myocardial cell apoptosis in rat heart failure model

**DOI:** 10.1590/acb396524

**Published:** 2024-09-30

**Authors:** Xiaofang Yang, Xuebin Zheng, Xiangqian Xiao, Li Li

**Affiliations:** 1 Changsha Fourth Hospital – Department of Cardiology – Changsha – Hunan – China.

**Keywords:** Heart Failure, Myocardium, Apoptosis

## Abstract

**Purpose:**

This work aimed to investigate the effects of Tanshinone IIA (Tan IIA) on myocardial cell (MC) apoptosis in a rat model of heart failure (HF).

**Methods:**

Tan IIA was extracted from *Salvia miltiorrhiza* Bunge (SMB) using an ethanol reflux method. Fifty rats were randomly divided into five groups: sham (no treatment), mod (HF model establishment), low dose (LD: 0.1 mL/kg Tan IIA), medium dose (MD: 0.3 mL/kg Tan IIA), and high dose (HD: 0.5 mL/kg Tan IIA), with 10 rats in each group. The effects of different doses of Tan IIA on cardiac function, MC apoptosis, and the levels of proteins associated with the PI3K/Akt/mTOR signaling pathway were compared.

**Results:**

Mod group showed a significant decrease in systolic arterial pressure, mean arterial pressure, heart rate, left ventricular systolic pressure, left ventricular ejection fraction, left ventricular fractional shortening, and the levels of p-PI3K, p-Akt, and p-mTOR proteins versus sham group (*p* < 0.05). Additionally, the left ventricular end-diastolic diameter (LVIDd), end-systolic diameter, diastolic pressure, and MC apoptosis were significantly increased (*p* < 0.05). LD, MD, and HD groups exhibited significant improvements across various indicators of cardiac function and MC apoptosis versus mod group (*p* < 0.05).

**Conclusions:**

Tan IIA may improve cardiac function and inhibit MC apoptosis in rats with HF by modulating the PI3K/Akt/mTOR signaling pathway.

## Introduction

With deepening of population aging, incidence of cardiovascular diseases (CVDs) has gradually increased, and heart failure (HF) represents the terminal stage of various CVDs^1^. HF leads to pathological changes in cardiac function, mainly characterized by ventricular remodeling, arrhythmias, and reduced ejection fraction^2^. Cell apoptosis is a common form of cell death, and myocardial cell (MC) apoptosis is a determining factor in the impairment of cardiac function^3^. With advancements in molecular biology, the role of signaling pathways (SPWs) in disease occurrence and development has garnered extensive attention. The PI3K/Akt/mTOR SPW participates in regulating MC apoptosis and powerfully influences HF^4^^,^ 5.

Traditional Chinese medicine treatment for HF is a safe and effective option. *Salvia miltiorrhiza* Bunge (SMB) is the dried root and rhizome of the plant SMB, which belongs to the Lamiaceae family and possesses functions. Modern pharmacological research has confirmed that active components in SMB can inhibit platelet aggregation, promote the fibrinolytic system, increase microcirculation capillary tension, and affect plasma viscosity, providing certain protective effects in myocardial ischemia and myocardial infarction^6^. SMB extract has a protective effect on the heart and blood vessels, exhibiting anti-arrhythmic, anti-atherosclerotic, and microcirculation-improving properties, which are beneficial for the adjunctive treatment of heart diseases^7^. SMB extract can also inhibit the activity of coronary artery platelets, thereby reducing platelet aggregation^8^. Additionally, SMB extract has a certain degree of inhibitory effect on the fibrinolytic system, effectively reducing hyperlipidemia and stabilizing atherosclerosis^9^.

Tanshinone IIA (Tan IIA) is the main lipophilic component in SMB extract, known for its blood circulation-promoting, analgesic, heat-clearing, and calming effects. Tan IIA can delay arterial hypoxia time, reduce hypoxia-induced damage to myocardial tissue, improve myocardial contractility, and promote the regeneration of MCs, playing a role in myocardial injury repair^10^. Moreover, Tan IIA can improve microcirculation by enhancing blood flow in capillaries and accelerating capillary dilation^11^. Cardiac fibrosis is a major cause of cardiac function (CF) impairment. Research has shown that SMB extract, Tan IIA, can reduce the level of myocardial tissue fibrosis^12^.

This work analyzed the *in-vivo* pharmacokinetic profile of SMB extract, evaluated its effects on CF and MC apoptosis in HF rats, and explored its relationship with the regulation of the PI3K/Akt/mTOR SPW, thus yielding a basis for clinical application of SMB extract in HF treatment.

## Methods

### Extract of active components from Salvia miltiorrhiza Bunge

The total of 5.5 mg of Tan IIA standard sample (HPLC ≥ 98%, obtained from Chengdu Preferred Biotechnology Co., Ltd., China) was precisely dissolved in 50 mL of methanol solution to obtain a 0.11 g/L stock solution of Tan IIA standard. Tan IIA was extracted from the SMB herbal pieces using an ethanol reflux method. Precisely weighed 100 g of SMB herbal powder was refluxed in a 95% ethanol solution for 1.5 h and then filtered. The residue was refluxed with two or four times the volume of 50% ethanol for an additional two hours and filtered. Designate the method with a 2x volume reflux at 50% as the EP-I method, and the method with a 4x volume reflux at 50% as the EP-II method. Water was added to the residue, and reflux was continued for two hours, followed by filtration. The filtrates from the second and third extractions were combined, and ethanol was recovered and concentrated to a relative density of 1.4. This was then mixed with the first concentrated solution, and ethanol was recovered and concentrated to obtain the extract.

### Pharmacokinetic assay on active components of Salvia miltiorrhiza Bunge extract

Six healthy male Sprague-Dawley rats (300 ± 10 g), each from Hunan SLAC Jingda Laboratory Animal Co., Ltd., China, were subjected to a 12-h fasting and water restriction period before the experiment. During the feeding, they had access to water during the experimental period. The extracted Tan IIA was administered through gavage. Six Sprague-Dawley rats were randomly divided into the EP-I group and the EP-II group, with three rats in each group. The EP-I group received intragastric administration of Tan IIA extracted using the EP-I method, while the EP-II group received Tan IIA extracted using the EP-II method. The administration was performed at the dose of 0.3 mL/kg. Blood samples of 0.5 mL were collected from the orbital venous plexus at 5, 15, 30, 60, 120, 240, 480, and 720 min post-administration, using heparin as an anticoagulant, and 0.2 mL of plasma was collected and mixed with 0.8 mL of acetonitrile. After mixing and vortexing for 2 min, the sample was arranged with 10-min centrifugation at 12,000 r/min for a collection of supernatants. Under the conditions of dry nitrogen at 37°C, the sample was dried, and 0.2 mL of the mobile phase was added to the residue. After vortexing for 2 min, the sample was again arranged with a 10-min centrifugation at 12,000 r/min. Twenty μL of supernatant were taken for analysis using the LabTech LC600 high-performance liquid chromatography system (Laidun Scientific Instruments Co., Ltd., Suzhou City, China). Differences in the pharmacokinetics of Tan IIA *in vivo* were compared after reflux filtration with different volumes of 50% ethanol.

All experiments were approved by Ethics Committee of Changsha Fourth Hospital, and in accordance with the Guide for the Care and Use of Laboratory Animals published by the United States National Institutes of Health.

### Preparation of rat heart failure model

A rat HF model was established using intraperitoneal injection of doxorubicin (Aureomycin). Forty Sprague-Dawley rats (6-week-old, weighing between 170 to 200 g, with an average weight of 188 ± 12 g) were adaptively fed for two weeks. Ten of these rats were randomly selected as the sham operation group, while the remaining 40 rats were used to create the HF model by intraperitoneal injection of 2 mg/mL doxorubicin (HPLC ≥ 98%, Jiangxi Baicaoyuan Biotechnology Co., Ltd., China) at the dose of 4 mg/kg, administered over six weeks. The sham operation group received an equivalent amount of 0.9% saline via intraperitoneal injection. After six weeks, rats were anesthetized with intraperitoneal injection of 1.5% sodium pentobarbital (HPLC ≥ 98%, New Asiatic Pharmaceutical Co., Ltd., China) at the dose of 30 mg/kg.

Once adequately anesthetized, the rats were placed in the supine position and fixed on the operating table, with the chest hair shaved. The left ventricular long-axis view was used for detection, and the left ventricular ejection fraction (LVEF) was measured using a high-resolution small animal ultrasound imaging system vevo2100 (Visualsonics, United States of America). An LVEF of less than 60% was considered indicative of a successful HF model.

### Different treatments of animals in various groups

The 40 successfully modeled rats were further grouped into four randomly: mod group, LD-, MD-, and HD-Tan IIA, each group consisted of 10 animals. In the LD-, MD-, and LD-Tan IIA groups, rats were subjected to intraperitoneal injection of 0.1, 0.3, and 0.5 mL/kg of Tan IIA extract suspension, respectively. In mod and sham groups, rats were administrated with an equal volume of physiological saline (PS) in an intraperitoneal injection way. All rats received weekly injections of the respective drugs for six consecutive weeks. During the treatment period, there were no reported rat deaths.

### Assessment of cardiac function

The rats were anesthetized with an intraperitoneal injection of 1.5% sodium pentobarbital, placed in the supine position, and securely fixed. The MD3000/8 Biological Function Experiment System Recorder (Anhui Zhenghua Biomedical Instrument Equipment Co., Ltd., China) was employed to measure the following indicators in rats: systolic arterial pressure (SAP), mean arterial pressure (MAP), heart rate (HR), and left ventricular systolic pressure (LVSP). Parameter settings were:

- Electrocardiogram: gain G500, time constant T 0.1 s, filter F300 Hz, scan speed 200 ms/div;
- Left ventricular pressure: gain G100, time constant T DC, filter F30 Hz, scan speed 200 ms/div;
- Left ventricular differential pressure: gain G100, time constant T DC, filter F10 Hz, scan speed 200 ms/div.

The ultra-high-resolution small animal ultrasound imaging system Vevo 2100 (VisualSonics, United States of America) with a 20-MHz high-frequency probe was used for M-mode echocardiography to detect the following indicators: LVEF, left ventricular fractional shortening (LVFS), left ventricular internal dimension diastole (LVIDd), left ventricular internal dimension systole (LVIDs), and left ventricular end-diastolic pressure (LVEDP).

### Myocardial histomorphology observation

After the CF measurement was completed, rats from each group were anesthetized with 30 mg/kg of 1.5% pentobarbital sodium intraperitoneal injection. Subsequently, they were euthanized by cervical dislocation, and the hearts were rapidly removed, excluding non-myocardial tissues. The myocardial tissue at the point of maximal ventricular diameter was trimmed into tissue blocks of size 1×1 cm. Some tissue blocks were soaked in 10% formalin solution for fixation for 24 hours, which were used for the preparation of paraffin sections. Another portion of the tissue was stored directly in liquid nitrogen. For the myocardial tissue fixed in 10% formalin solution, routine gradient dehydration, transparency, and paraffin embedding were performed to prepare myocardial tissue paraffin sections. Four-μm thick paraffin sections were obtained and subjected to deparaffinization, hydration in ethanol, and washing. They were then gradually stained using Sudan III and eosin staining solution (Shanghai Jinsui Biotechnology Co., Ltd., China), excess stain was washed off, and the sections were mounted with neutral gum. The morphological changes in myocardial tissue were observed, which was realized using an Olympus CX23 upright biological microscope (Olympus, Japan).

### Histopathological observation of myocardial tissue

Hematoxylin and eosin (HE) staining was used to observe the histopathology of myocardial tissue in each group of rats. After euthanasia, the myocardial tissue was collected and fixed with 4% paraformaldehyde solution for 30 min. The samples were then dehydrated with ethanol, embedded in paraffin, and continuously sectioned using a microtome. The sections were sequentially placed in the following solutions: Xylene I for 10 min, Xylene II for 10 min, absolute ethanol I for 5 min, absolute ethanol II for 5 min, 95% ethanol for 5 min, 90% ethanol for 5 min, 80% ethanol for 5 min, 70% ethanol for 5 min, and then washed with distilled water. Then, the sections were stained with Harris hematoxylin for 3–8 min and rinsed with tap water. Differentiation was performed using 1% hydrochloric acid alcohol for a few seconds, followed by washing with tap water. The sections were then blued with 0.6% ammonia water and rinsed with running water. Eosin staining solution was applied for 1–3 min. The sections were sequentially dehydrated in 95% ethanol I for 5 min, 95% ethanol II for 5 min, absolute ethanol I for 5 min, absolute ethanol II for 5 min, Xylene I for 5 min, and Xylene II for 5 min. After clearing in xylene, the sections were slightly dried and mounted with neutral balsam. Finally, the sections were observed under a microscope, and images were captured and analyzed.

### Detection of myocardial cell apoptosis

Four-μm thick paraffin sections were subjected to terminal deoxynucleotidyl transferase dUTP nick end labeling (TUNEL) staining reagent kit (Shanghai Beyotime Biotechnology Co., Ltd., China) and placed in 4% paraformaldehyde solution and incubated at room temperature (RT) for half an hour. Deparaffinization was performed using xylene, followed by tissue dehydration with a gradient of ethanol solutions. The sections were then processed with a 0.5% Triton X-100 at RT for 5 min. After that, prepared TUNEL labeling solution was mixed to allow a 1-h centrifugation without light at 37°C. Subsequently, 2-(4-Amidinophenyl)-6-indolecarbamidine dihydrochloride (DAPI) staining solution (Shanghai Beyotime Biotechnology Co., Ltd., China) was added to allow an incubation lasting for 5 min. Eventually, these sections were sealed with anti-fluorescence quenching mounting solution, and MC apoptosis was observed using an OLS5100 Laser Confocal Microscope (Olympus, Japan). Image J was utilized to calculate the MC apoptosis index.

### Determination of proteins related to PI3K/Akt/mTOR SPW

The tissues stored in liquid nitrogen were taken out and thoroughly ground. Radioimmunoprecipitation assay buffer (ThermoFisher, United States of America) was added to fully dissolve the tissues and extract the proteins, with the concentration being determined using Bicinchoninic acid protein quantification assay kit (Shanghai Beyotime Biotechnology Co., Ltd., China). Fifteen μg of protein was loaded and transferred to a polyvinylidene difluoride (PVDF) membrane for 90 min, followed by a block with 5% non-fat milk at RT for 2 hours. Primary antibody (Abcam, United Kingdom) PI3K, p-PI3K, Akt, p-Akt, mTOR, p-mTOR, and glyceraldehyde 3-phosphate dehydrogenase (GAPDH) were added at a 1:1,000 dilution ratio, and the membrane was incubated all the night at 4°C. After washing, the membrane was added with a secondary antibody (Abcam, United Kingdom) diluted at a 1:2,000 ratio for an incubation at RT for two hours. Protein bands were visualized using an enhanced chemiluminescence kit (ThermoFisher, United States of America). Eventually, after imaging with the Tanon 1,600 Gel Imaging System (Shanghai Tanon Technology Co., Ltd., China), protein expression relative grayscale values were quantified using image processing and analysis in Java 2 (ImageJ 2).

### Statistical analysis

Data were statistically analyzed using Statistical Package for the Social Sciences (SPSS) 22.0. All data followed a normal distribution and were presented as mean ± standard deviation. One-way analysis of variance was utilized for intergroup comparisons, and Fisher’s least significant difference (LSD) test was employed for comparisons between two groups. *p* < 0.05 was considered statistically significant.

## Results

### In-vivo pharmacokinetic analysis on active components of Salvia miltiorrhiza Bunge extract

**Figure 1:**
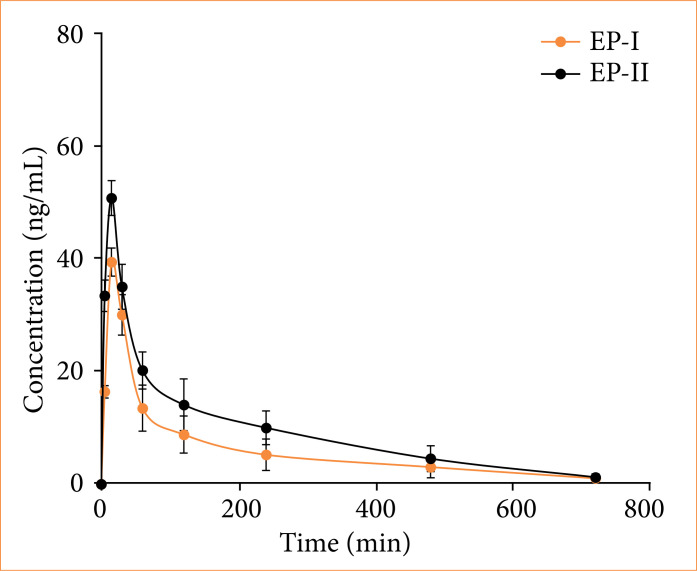
*In-vivo* BDC-time curve of Tan IIA with distinct extraction methods.

BDC: blood drug concentration; Tan: Tanshinone. Source: Elaborated by the authors.

**Figure 2:**
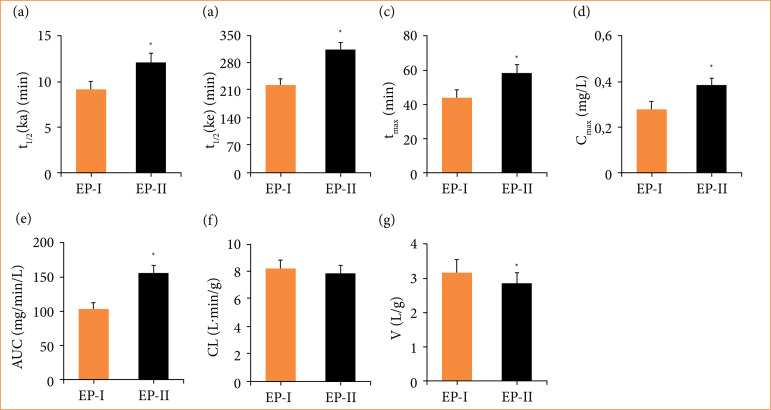
*In-vivo* pharmacokinetic parameters of Tan IIA under different extraction methods. **(a)** Absorption half-life; **(b)** elimination of half-life; **(c)** peak blood drug concentration; **(d)** time to peak blood drug concentration; **(e)** area under the blood drug concentration-time curve; **(f)** clearance rate; **(g)** drug dose/blood drug concentration ratio; EP-I and EP-II referred to the extraction method I with two-fold and four-fold volume, respectively.

In this work, Tan IIA from SMB was extracted, and impacts of different extraction methods on the pharmacokinetics of Tan IIA were examined. The blood-drug concentration (BDC)-time curve was presented in Fig. 1, and the pharmacokinetic parameters were compared in Fig. 2. In contrast to EP-I, EP-II extraction method greatly increased t_1/2_(ka), t_1/2_(ke), T_max_, C_max_, and AUC, while sharply decreasing the V parameter, showing great differences (*p* < 0.05).

Tan: Tanshinone; *significant difference with *p* < 0.05 to the EP-I group. Source: Elaborated by the authors.

### Impacts of Salvia miltiorrhiza Bunge extract on rats with heart failure

**Figure 3:**
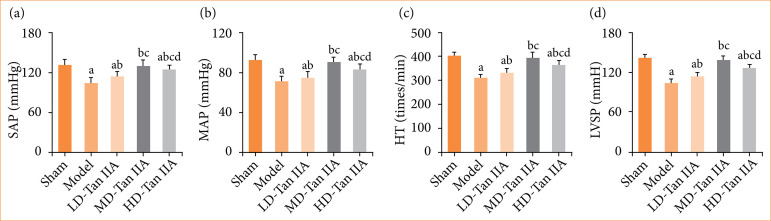
CF-related parameters of rats with heart failure. (a–d): SAP, MAP, HR, and LVSP, respectively. There was significant difference with *p* < 0.05 in comparing with sham, mod, LD-, and MD-Tan IIA group, respectively.

SAP: systolic arterial pressure; MAP: mean arterial pressure; Tan: tanshinone; LD: low density; MD: medium density; HD: high density; HR: heart rate; LVSP: left ventricular systolic pressure; CF: cardiac function. Source: Elaborated by the authors.

This work evaluated the impact of Tan IIA on cardiac function in HF rats. As demonstrated in Fig. 3, mod group showed reduced SAP, MAP, HR, and LVSP, presenting considerable differences based on the sham group (*p* < 0.05). In comparison to the mod group, the LD-, MD-, and HD-Tan IIA groups exhibited visible increases in SAP, MAP, HR, and LVSP indices (*p* < 0.05), with the LD-Tan IIA group having the lowest values and the HD-Tan IIA group holding the highest values (*p* < 0.05). It indicates that Tan IIA has a positive impact on CF in HF rats, as evidenced by its ability to increase SAP, MAP, HR, and LVSP, thereby improving the CF.

**Figure 4:**
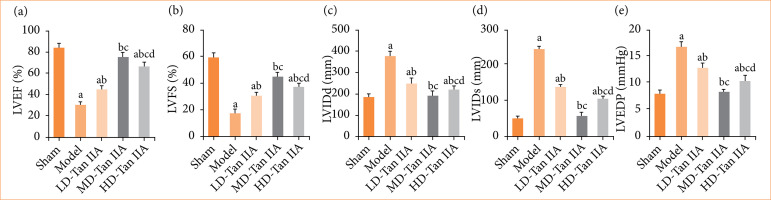
M-mode echocardiography results of heart failure rats. (a–e): LVEF, LVFS, LVIDd, LVIDs, and LVEDP, respectively. (a–d) significant difference with *p* < 0.05 in comparing with sham, mod, LD-, and MD-Tan IIA group, respectively.

LVEF: left ventricular ejection fraction; LVFS: left ventricular fractional shortening; LVIDd: left ventricular internal dimension diastole; LVIDs: left ventricular internal dimension systole; LVEDP: left ventricular end-diastolic pressure; Tan: tanshinone; LD: low density; MD: medium density; HD: high density. Source: Elaborated by the authors.

As illustrated in Fig. 4, the mod group exhibited greatly reduced LVEF and LVFS, but increased LVIDd, LVIDs, and LVEDP, showing observable differences with *p* < 0.05. The three Tan IIA groups presented increased LVEF and LVFS, but decreased LVIDd, LVIDs, and LVEDP in contrast to the mod group, with considerable differences (*p* < 0.05). The LD-Tan IIA group possessed the highest LVEF and LVFS, as well as the lowest LVIDd, LVIDs, and LVEDP among the three Tan IIA treatment groups (*p* < 0.05). Furthermore, LVEF and LVFS were lower, and LVIDd, LVIDs, and LVEDP were higher in the HD-Tan IIA group, showing remarkable differences with those in MD-Tan IIA group (*p* < 0.05).

### Impacts of Salvia miltiorrhiza Bunge extract on heart histopathologic parameters of heart failure rats

**Figure 5:**
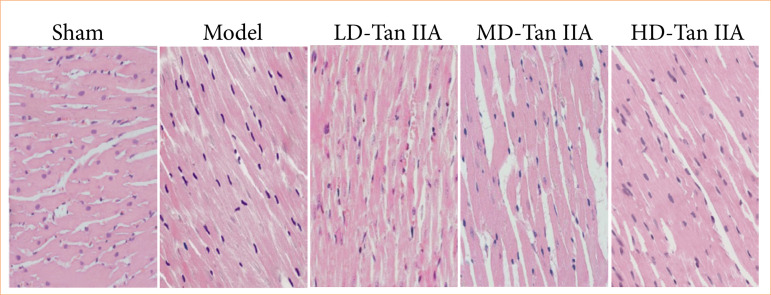
Hematoxylin and eosin staining results of myocardial tissues of heart failure rats (×200).

In this work, myocardial tissue sections were prepared and stained with HE. As summarized in Fig. 5, the myocardial tissue of rats appeared normal with clear texture, and no cell necrosis or fibrosis was observed in rats after sham. However, in mod group, there were patches of cell necrosis, partial dissolution of MCs, and interstitial fibrosis. In the three Tan IIA treatment groups, the phenomenon of patchy necrosis in myocardial tissue improved, and fibrosis was alleviated.

Tan: tanshinone; LD: low density; MD: medium density; HD: high density. Source: Elaborated by the authors.

### Impacts of Salvia miltiorrhiza Bunge extract on myocardial cell apoptosis of heart failure rats

**Figure 6:**
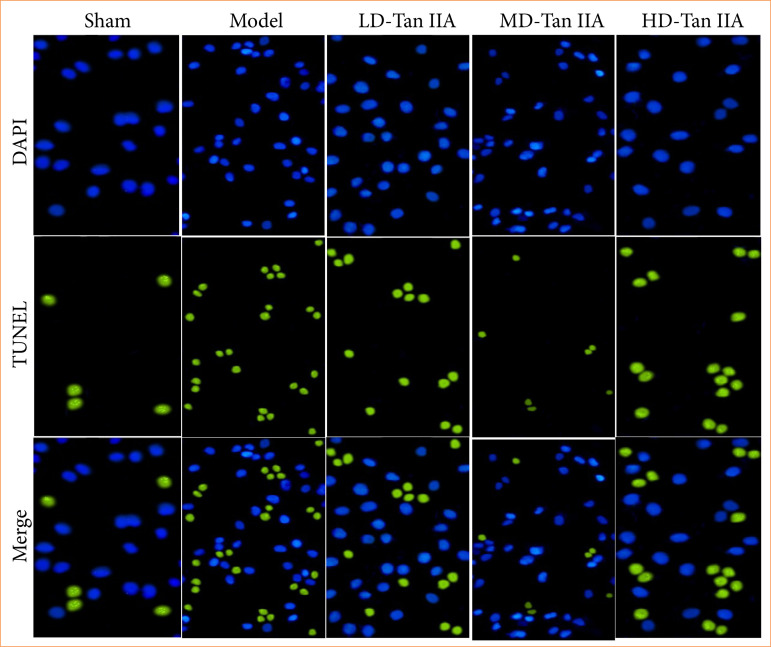
TUNEL staining results of myocardial tissues of heart failure rats (×200).

**Figure 7:**
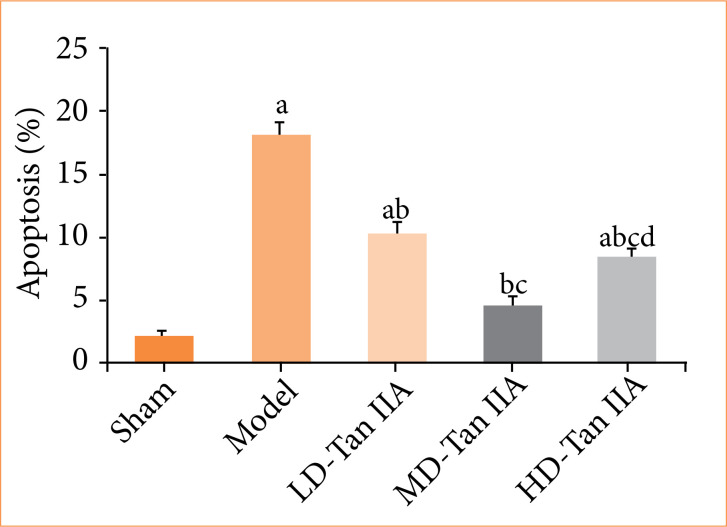
Myocardial cell apoptosis of rats with heart failure. (a–d) indicated significant difference with *p* < 0.05 in comparing with sham, mod, LD-, and MD-Tan IIA group, respectively.

As explicated in Fig. 6, apoptotic MCs were stained with green fluorescence, and MCs with green fluorescence in the mod group were more based on those in sham group. Figure 7 displayed the detection of MC apoptosis. MC apoptosis was sharply increased in the mod group, with an observable difference to the sham group (*p* < 0.05). MC apoptosis was decreased in the Tan IIA treatment groups, presenting obvious differences with that in rat HF model (*p* < 0.05). The LD-Tan IIA group exhibited higher MC apoptosis among the three Tan IIA treatment groups (*p* < 0.05). In contrast to the MD-Tan IIA group, MC apoptosis was increased in HD-Tan IIA group, with an obvious difference (*p* < 0.05).

Tan: tanshinone; LD: low density; MD: medium density; HD: high density; DAPI: 2-(4-Amidinophenyl)-6-indolecarbamidine dihydrochloride; TUNEL: terminal deoxynucleotidyl transferase dUTP nick end labeling. Source: Elaborated by the authors.

Tan: tanshinone; LD: low density; MD: medium density; HD: high density. Source: Elaborated by the authors.

### Impacts of Salvia miltiorrhiza Bunge extract on PI3K/Akt/mTOR SPW of heart failure rats

**Figure 8:**
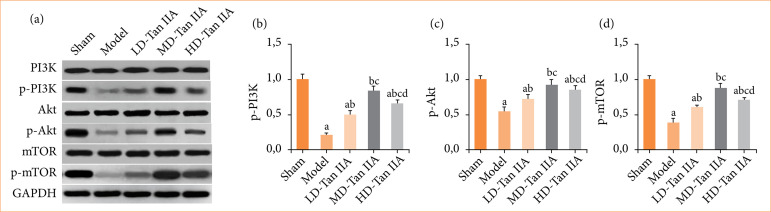
Expressions of PI3K/Akt/mTOR SPW-related proteins in heart failure rats. **(a)** Western blot results; (b–d): p-PI3K, p-Akt, and p-mTOR, respectively. (a–d) Significant difference with *p* < 0.05 in comparing with sham, mod, LD-, and MD-Tan IIA group, respectively.

In this work, changes in PI3K/Akt/mTOR SPW-related proteins were examined. As explicated in Fig. 8, p-PI3K, p-Akt, and p-mTOR in mod group were greatly downregulated, presenting obvious differences with those in sham group (*p* < 0.05); those in mod group were lower to those in three Tan IIA treatment groups (*p* < 0.05); and those in LD-Tan IIA group were the lowest among all Tan IIA treatment groups (*p* < 0.05). Furthermore, above indicators in the cardiac tissue of the HD-Tan IIA group were decreased, showing obvious differences with those in MD-Tan IIA group (*p* < 0.05).

Tan: tanshinone; LD: low density; MD: medium density; HD: high density; GAPDH: glyceraldehyde 3-phosphate dehydrogenase. Source: Elaborated by the authors.

## Discussion

SMB is a medicinal herb known for its blood-activating and stasis-removing properties, promoting blood circulation, relieve pain, clear the mind, and cool blood to resolve abscesses^13^. SMB ketone is a lipid-soluble quinone compound extracted from SMB, which has antibacterial effects. It includes SMB ketone I, Tan IIA, SMB ketone IIB, and cryptotanshinone. SMB ketone has antibacterial, anti-inflammatory, blood-activating, and wound healing properties^14^^–^ 16. Tan IIA, a sulfonated product of SMB ketone, is water-soluble and has been clinically proven to be effective in treating angina with minimal side effects, making it a promising new drug for coronary heart disease^17^^,^ 18. Furthermore, recent theoretical studies and clinical practices have demonstrated that SMB ketone compounds also possess certain anti-tumor activities and can significantly inhibit liver cancer cells, leukemia cells, gastric cancer cells, and others^19^^,^ 20.

In this work, Tan IIA was extracted from *Salvia miltiorrhiza*, and its pharmacokinetic parameters were evaluated. It was observed that, after purification of the Ep-II extract using four-fold volume of 50% ethanol reflux, parameters such as t_1/2_, C_max_, and AUC were increased. t_1/2_ generally refers to the drug half-life, which is the time for drug concentration in blood or amount of the drug to decrease by half^21^. Tmax is the time to reach the maximum BDC value on the concentration-time curve, representing the maximum concentration achieved after drug administration^22^. Cmax refers to the time for reaching the maximum value of drug in human body after administration^23^. AUC represents the bioavailability of a drug, which reflects the extent of drug absorption and utilization in the body. A higher AUC indicates better bioavailability, and viceversa^24^. Results obtained in this work confirmed that the EP-II method provides better pharmacokinetic properties of Tan IIA compared to EP-I, and it has improved bioavailability.

HF is characterized by impaired ventricular diastolic or systolic function, with arterial ischemia and/or venous congestion as the main feature^25^. Recently, incidence, mortality, and readmission rates of HF have remained high, posing a serious threat to patients’ lives and health. SMB promotes blood circulation and improves microcirculation and enhance cardiac contractile function^26^. The application of SMB in HF can improve heart failure symptoms and increase myocardial contractility^27^. Tan IIA is extracted from SMB, accounting for about 0.1 to 0.9% of the SMB herb^28^.

The present work revealed that, following treatment with Tan IIA, cardiac function indicators including SAP, MAP, HR, and LVSP in HF rats were all higher than those in HF rats without treatment. Moreover, M-mode echocardiography suggested that LVEF and LVFS in HF rats increased after treatment, while LVIDd, LVIDs, and LVEDP decreased. The MAP index can effectively reflect cardiac pump function and is commonly used in CVDs prediction and prognosis assessment. LVSP is an important cardiac function index, reflecting changes in left ventricular pressure and volume during cardiac contraction^29^. LVEF is an effective indicator for assessing HF, as it is closely related to myocardial contractility, with lower LVEF values indicating weaker myocardial contractile force^30^.

Previous research has demonstrated that Tan IIA can inhibit platelet aggregation and hinder thrombus formation *in vitro* 31. Tan IIA can regulate microtubule acetylation and inhibit extracellular signal-regulated kinase 2 phosphorylation, exerting anticoagulant effects^32^. Microcirculatory disorders are associated with changes in blood rheological properties, leading to vascular stenosis, decreased blood flow velocity, or thrombus formation^33^. Tan IIA can improve microcirculatory disorders in disseminated intravascular coagulation and lymphatic microcirculation^34^. Thus, it is confirmed that Tan IIA can partially improve cardiac function in HF rats, although its mechanism of action requires further investigation.

Myocardial fibrosis is the consequence of fibrous connective tissue affecting the heart and is crucial in HF. Prolonged pressure overload leads to excessive deposition of extracellular matrix proteins in cardiac tissue, causing pathological remodeling of the interstitium, which results in increased myocardial stiffness, reduced myocardial compliance, and impaired cardiac contraction and relaxation functions^35^^–^ 37. The results confirmed that, after treatment with Tan IIA, the degree of fibrosis in the myocardial tissue of HF rats significantly decreased, and there was also an improvement in the patchy necrosis phenomenon within the myocardial tissue. Tan IIA exerts an anti-liver fibrosis effect by protecting hepatocytes, regulating hepatic stellate cell activity, removing extracellular matrix, and improving microcirculation^38^. Moreover, Tan IIA can lower the expression of nicotinamide adenine dinucleotide phosphate (NADPH) oxidase subunits and reactive oxygen species levels, thereby reducing myocardial fibrosis^39^. Results herein indicate that Tan IIA can alleviate cardiac fibrosis in HF rats.

MC apoptosis is a crucial factor in the progression of HF and a major characteristic of HF at the terminal stage. In the early stages of increased cardiac pressure load, apoptosis of MCs leads to compensatory enhancement. With the continuous increase in cardiac pressure load, the number of apoptotic MCs also greatly increases, and MCs tend to die in the balance between proliferation and apoptosis, resulting in a progressive decrease in the number of MCs^40^^,^ 41. Tan IIA can reverse the downregulation of miR-133, inhibit the cascade induction of caspase-9 and related apoptotic effectors, thereby significantly improving H_2_O_2_ or doxorubicin-induced MC apoptosis and myocardial injury^42^.

Additionally, the results demonstrated that the number of TUNEL-positive stained cells in myocardial tissue significantly reduced after Tan IIA treatment, indicating a decrease in the rate of cellular apoptosis within the myocardial tissue. The mentioned findings indicate that Tan IIA can inhibit MC apoptosis in HF rats, thereby improving CF in the rats.

PI3K, a member of phosphoinositide 3-kinase family, serves as an intracellular lipid kinase associated with oncogenic products like v-src and v-ras. It specifically catalyzes the phosphorylation of phosphoinositides at the three-position hydroxyl, generating phosphoinositide lipid kinases that act as second messengers, recruiting and activating downstream target molecules to initiate a series of signaling cascades^43^. Akt is one downstream target of PI3K, and it regulates various cellular processes. PI3K/Akt/mTOR SPW is important in vascular formation. It has been confirmed that, under pressure overload, blocking the vascular endothelial growth factor (VEGF) will decrease capillary density and accelerate the transition from compensatory cardiac hypertrophy to heart failure^44^. If the Akt is activated for a long time, it can lead to pathological cardiac hypertrophy, downregulated VEGF and vascular endothelial growth factor-2, significant decrease in capillary density, and regulation of endothelial cell migration, thereby influencing the progression of heart failure^45^. MC apoptosis is a major feature of HF, and the PI3K/Akt/mTOR SPW can directly or indirectly inhibit the effect of apoptosis factors^46^.

Additionally, the PI3K/Akt/mTOR SPW can influence HF progression through regulating metabolism, calcium cycling proteins, and inflammatory responses^47^. The results confirmed that, after treatment with Tan IIA, the expression levels of PI3K/Akt/mTOR pathway-related proteins p-PI3K, p-Akt, and p-mTOR in myocardial tissue of HF rats all significantly decreased, indicating inhibition of PI3K/Akt/mTOR pathway activation. Tan IIA has a protective effect on vascular endothelial cells and can also inhibit atherosclerosis^48^. These results indicate that Tan IIA can regulate the PI3K/Akt/mTOR SPW activation, exerting its role in regulating HF and MC apoptosis.

## Conclusion

The four-fold ethanol volume extraction of Tan IIA from SMB resulted in superior *in-vivo* pharmacokinetic levels. Moreover, Tan IIA effectively enhanced the CF of HF rats, inhibited MC apoptosis, and mediated the PI3K/Akt/mTOR SPW. However, this work only assessed the therapeutic effects of distinct concentrations of SMB extract in treating HF, without comparing them to positive drugs for treatment efficacy. Additionally, the long-term therapeutic effects of SMB extract in treating HF were not evaluated. Further research is needed to investigate the effects of SMB extract in treating HF in the future.

## Data Availability

The data will be available upon request.
